# Designing faculty development: lessons learnt from a qualitative interpretivist study exploring students’ expectations and experiences of clinical teaching

**DOI:** 10.1186/s12909-019-1480-7

**Published:** 2019-02-07

**Authors:** Julia Blitz, Marietjie de Villiers, Susan van Schalkwyk

**Affiliations:** 1 Centre for Health Professions Education, Faculty of Medicine and Health Sciences, Stellenbosch University, Stellenbosch, South Africa; 2 Division of Family Medicine and Primary Care, Faculty of Medicine and Health Sciences, Stellenbosch University, Stellenbosch, South Africa

**Keywords:** Faculty development, Clinical teachers, Student experience, Agency, Engagement, Workplace-based learning

## Abstract

**Background:**

Clinical teaching plays a crucial role in the transition of medical students into the world of professional practice. Faculty development initiatives contribute to strengthening clinicians’ approach to teaching. In order to inform the design of such initiatives, we thought that it would be useful to discover how senior medical students’ experience of clinical teaching may impact on how learning during clinical training might be strengthened.

**Methods:**

This qualitative study was conducted using convenience sampling of medical students in the final two months of study before qualifying. Three semi-structured focus group discussions were held with a total of 23 students. Transcripts were analysed from an interpretivist stance, looking for underlying meanings. The resultant themes revealed a tension between the students’ expectations and experience of clinical teaching. We returned to our data looking for how students had responded to these tensions.

**Results:**

Students saw clinical rotations as having the potential for them to apply their knowledge and test their procedural abilities in the environment where their professional practice and identity will develop. They expected engagement in the clinical workplace. However, their descriptions were of tensions between prior expectations and actual experiences in the environment.

They appreciated that learning required them to move out of their “comfort zone”, but seemed to persist in the idea of being recipients of teaching rather than becoming directors of their own learning. Students seem to need help in participating in the clinical setting, understanding how this participation will construct the knowledge and skills required as they join the workplace. Students did not have a strong sense of agency to negotiate participation in the clinical workplace.

**Conclusions:**

There is the potential for clinicians to assist students in adapting their way of learning from the largely structured classroom based learning of theoretical knowledge, to the more experiential informal workplace-based learning of practice. This suggests that faculty developers could broaden their menu of offerings to clinicians by intentionally incorporating ways not only of offering students affordances in the clinical learning environment, but also of attending to the development of students’ agentic capability to engage with those affordances offered.

**Electronic supplementary material:**

The online version of this article (10.1186/s12909-019-1480-7) contains supplementary material, which is available to authorized users.

## Background

It is in the clinical environment that students learn what it means to be a “real doctor” [1] with opportunities to apply their newly acquired medical knowledge to patient care [2]. Learning in the clinical setting constitutes an important part of the medical curriculum, particularly in the senior years when it is seen as the culmination of preparation of the student for internship and a prelude to independent practice. Undergraduate medical curricula, therefore, are often rounded off with a student internship (clerkship) as the final phase. This liminal space [3] provides the opportunity to make use of the support and supervision characteristic of being a student, while learning the tasks and responsibilities that will be required after graduation. In order to use the opportunity optimally, the student is required to adapt their way of learning from the largely structured classroom based learning of theoretical knowledge to the more experiential informal workplace-based learning of practice [4, 5].

Billett has developed an argument around what might be necessary for learning to happen in the workplace. His work has progressed from a call for practice settings (and not only educational institutions) to be legitimated as environments which make a contribution to the learning of people who participate and learn in them [6], through recognising that support of that learning is mediated by the kinds of activities in which learners engage [7] to an understanding that this requires learners to utilise everyday work activities as pedagogically rich opportunities [8]. The emphasis on the social nature of learning emerges clearly in his thinking. As learning requires the learner to be involved and consciously engaged, the priority in creating learning in the workplace becomes the supervisor’s ability to promote effective engagement; the clinical supervisor needs to afford the learner the opportunities [9]. The similarity with situated learning can be seen, through which participation in meaningful work facilitates the learner joining the community of practice [10]. This is where the specific learning potential of the student internship lies.

Although many clinical teachers are enthusiastic and can be assumed to have clinical competencies, they may lack the knowledge of educational principles and teaching strategies (communication, adult learning principles, use of new information technology, etc.) to use in a healthcare system that may be quite transformed from that in which they were trained [11, 12]. Research has shown that performance of students is related to the competence of their teachers [13]. Dornan’s [9] and Billett’s [14] work suggests that the way clinical teaching is carried out will have consequences for how students learn and understand, with implications for whether the student is fully able to benefit from their clinical training time [15, 16]. All this points to the need to support clinical teachers in this particular teaching role.

This article presents one of four components of a situational analysis of clinical teaching, which was conducted in order to inform strengthening of faculty development initiatives for clinical teachers. This component, set out to understand students’ perspectives on their expectations and experiences of clinical learning.

## Methods

As the goal of this study was generating rich perspectives on the experiences of students, a qualitative approach was chosen within an interpretivist paradigm [17, 18].

At the time this research was conducted, the researchers had various responsibilities for contributing to the quality of clinical teaching provided by this faculty: JB is a family physician who was responsible for educational capacity development; MdV is a family physician who was responsible for quality assurance of undergraduate education; SvS is an educationalist contributing to the professionalisation of health professions education. We have been involved in a variety of research projects looking at a range of aspects with regard to the educational impact of the faculty’s rural clinical school on the academic programme, staff and students [19–21]. None of us were involved in the teaching programme of these students.

At this institution, each class of medical students is randomly divided into four groups (approximately 50 students per group) for the last eighteen months of their programme, to do rotations (clerkships) in a number of clinical disciplines and at a variety of clinical teaching sites, some of which are outside the traditional teaching hospital. This research was conducted in the last two months prior to their graduation. By that point in time the class was completing their final rotation and would therefore all have had a similar range of experiences across the clinical training platform. Convenience sampling was used to identify students in three rotations that were accessible at the time planned for data collection. All students in these rotations were informed about the research by the researcher, given informed consent forms and requested to return completed forms to the research assistant. Those students who gave consent were then invited to participate in a focus group discussion with the other consenting members of their rotation group. This method was chosen for its fit with research conducted in the constructivist paradigm, particularly as this was an issue that individual students may have felt less free to explore; specifically we wanted to use the dynamics between students in the group to “prompt fuller and deeper discussion and the triggering of new ideas” [22], and “capitalize on the synergy arising from interactions of the members” [23].

Three focus group discussions were held with a total of 23 students (8 males, reflecting the demographics of the faculty’s student body) and facilitated by the lead researcher. The discussions lasted between 70 and 80 min. An interview guide covered the following areas which the researchers thought would have the potential to inform ways of strengthening clinical teaching (see Additional file 1 for prompt questions):

- how student interns experience clinical rotations, in particular what they identify as clinical teaching;
- what clinical teaching practices they consider to have been of the greatest benefit to their learning;
- suggestions that they have for the improvement of clinical teaching.

In order to promote trust and confidentiality, individuals were not identified by name, only by a letter of the alphabet. Participants in the focus group discussion agreed to maintain the confidentiality of the discussions. The discussions were audio-recorded and then transcribed verbatim. The transcriber signed a confidentiality agreement. Ethics approval was obtained from the Stellenbosch University Health Research Ethics Committee (#N14/08/097).

Following an interpretive approach, thematic analysis was performed [24, 25] inductively using Atlas.ti to obtain a clearer understanding of the students’ perspectives on how clinical teaching could be strengthened. Braun and Clarke’s suggested criteria for good thematic analysis were used during both coding and analysis phases. Trustworthiness and credibility were attended to [17] by addressing craftsmanship; iteratively checking, questioning and theoretically interpreting the findings. The initial coding and generation of themes was done by JB. MdV and SvS each read one of the transcripts and as a team we then discussed and reached agreement on the final themes.

## Results

Aligned with our interpretivist stance, the results section initially presents the themes that we developed from the data as our interpretation of these as tensions between the students’ prior expectations of clinical teaching and their actual experience thereof. While analysing the data we continually referred to how the findings would impact on faculty development initiatives to strengthen the teaching role of clinicians - the action that would result from the research. Therefore, having identified these tensions, we went on to a second round of deeper analysis to understand how students chose to respond (or not) to them. Our interpretations of their responses are presented in the latter part of this results section.

There was a mixture of experiences – for some individuals their experience had been of met expectations, reinforcing their beliefs that these were indeed reasonable expectations. However, we also heard of clinical experiences in which expectations were unmet, playing out as tensions between actual experiences in the light of preceding expectations (Table 1).

**Table 1:** Depicts tensions between students’ actual experiences and their preceding expectations

Students experience was that … …	despite having expected that … …
only selected clinicians were enthusiastic about teaching responsibilities, … …	… … skilled teaching is what professionals should do.
they seldom have the opportunity to observe clinicians in action, … …	… … there would be opportunities to do so.
it isn’t always easy to become a member of the clinical team, … …	… … they would join clinical teams.
clinicians did not have time to interrogate clinical reasoning, … …	… … this was the time when they would be able to test their clinical reasoning skills.
they are very seldom observed and even less often given feedback, … …	… … they would receive corrective feedback on their performance.
the complexity of clinical teaching was frustrating so they prefer to be given the “correct” answers, … …	… … learning would be facilitated by being given responsibility for patient care.
it is difficult to ask for teaching, … …	… … clinical teaching is about opportunities to “do”.

Students expected teaching to be a component of all doctors’ (including registrars’) obligations, **but** experienced that not all clinicians embraced this professional role.

*I feel like the whole concept that medicine is sort of taught from the most experienced downwards, it’s the best way, and I think that some people don’t think about it that way. … So “I am here to get my degree and I don’t care about anyone that is rotating under me, or the gap that is forming in their knowledge because I'm not willing to give some of my time”* [voicing a perception of the registrar’s attitude]*. That mentality to be able to give back because you have been privileged enough to get is not present in all.*

[F]

Students had thought that they would learn by seeing clinicians in action, **but** such occasions did not necessarily translate into learning opportunities.

*I also think it’s kind of rushed. So even if you get to sit in with the doctor during clinic for instance, again, they’re not doing things systematically. They are doing things by* [sic] *their own, so it’s not exactly a good place to pick up.*

*[FS2]*

Students found that **despite** identifying the need to do so, they were not always invited to join a clinical team or given responsibility.

*… immediately with that first contact you can tell if they’re like … “just go and work there”, or if they say “hey, welcome to the team, we work in this, this and this ward, can you see patients there, if you have any questions come to me”. It’s very different.*

[FS]

*[being part of the team] gives you confidence, and you need confidence to be out there on your own, in your abilities and your skill.*

*[FS2]*

*I think the one doctor where we learnt the most is one where he gave us a patient, told us go across the corridor, see a patient on your own, call me when you are ready, and by that time we would have discussed everything that might be a possible diagnosis, read up about everything. We are not going to be asked a question and not be able to answer, and then afterwards he will teach us and give us practical tips, like pearls … . I think that is also, that comes in with being thrown into the deep side, being a little bit uncomfortable. Like you don’t really know what you are doing now, but it’s still a safe way of not knowing what you are doing … . Because it’s very easy to stagnate behind the doctor’s back while he’s doing the work and you are just like trying to read what he is writing. I don’t think that is the optimum way to learn in the hospital. I think it’s by being in the frontline. … It’s almost like he gave us responsibility for that patient.*

[E]

**Although** students anticipated that they would have opportunities to apply their knowledge to real patient care, clinicians did not always have the time to explore students’ clinical reasoning.

*I feel that in hospital, in bedside teaching, ward rounds … . It’s as if it is integrated, because you can see the pathology lying there, and then they often teach you an approach like you can divide the causes up into a few groups. So then you learn to think about stuff in a logical and structured way, and that way you remember it. You are not going to remember it by regurgitating a list over and over and over.*

[E]

*take you right from your whole presentation, why are you saying this now, why you say it here. So really help you sort of construct a nice approach to how to present a patient, and then to go and get you to examine the patient in front of them. How do you check for that reflex and why do you say this, and listen here. Not every time, because it is time consuming, but maybe just once a week to try with each student.*

*[O]*

Students expected feedback on their performance, **but** reported that they were very seldom observed and even less often given feedback.

*there is just too little time. The physicians or the surgeons or whatsoever don’t have all the time to observe. … they will send the intern with you. The intern is also qualified, they can do it, but you don’t get the expert watching you do it.*

[A]

*I want somebody to teach me the things that I don’t know, or the things that I am missing, or to correct my...*

[FS]

*… they don’t always know that we are okay if you tell us that you have done the wrong thing. That’s fine, just tell me what to do. It’s like you don't want to step on toes, … . It‘s not harmful to us, it’s fine, we’re okay, but just to give doctors that permission that they can tell students and redirect, that’s fine.*

[V]

*At least explain why this is wrong.*

[H]

Students found the complexity of clinical care frustrating and **thus** resorted to wanting the “answers”.

*In the beginning I felt so frustrated, because whenever you ask them a question, they give you a question back. … , what I mean is you want an answer because you think that that is the best way. You want an answer. What are the causes? This is the list, you want that answer.*

[F]

*… it’s so much more useful to get an answer from the doctor that exactly knows, that has been through all of this a thousand times before, to make sense of it and now give it to us.*

*[D]*

**Despite** expecting that clinical teaching was about “doing”, students found it difficult to ask to be taught in the clinical environment.

*… if they are all busy and running around, … you’re not going to be like oh, can you please sit down and talk about something.*

[O]

*You don’t want to inconvenience anyone*

[N]

*because I don’t know you and it’s the first time I'm working with you, I am intimidated and I see you as I'm just going to be an irritation, so let me rather reserve myself until you maybe come forward or offer to teach, and then only will I be willing to ask.*

[I]

*I always feel guilty asking the doctor to spend extra time with me now to go and practice or do this thing, because I know it’s going to take him twice as long, and it might hurt the patient more if I try it the first time. It’s a difficult place to be.*

[G]

Having found these tensions we returned to the data looking for how students had responded to that tension between expectation and experience.

Students realised that learning was often accompanied by being a bit uncomfortable.

*At the end of the day, it comes down to you remain the student. There is nothing you can do about it. … be aware of the fact that we are sometimes scared to approach them*

[L]

*… it will make it more comfortable for us to be willing to ask questions, even if we feel like the doctor isn't approachable. It will put us in a situation where we can.*

[I]

*So you’ve just got to sort of grow.*

[O]

However, they also acknowledged that as they approached graduation, they forced themselves to be involved, realising there was a real need to be able to “do” as they started anticipating their role as an intern.

*Now in final year, I don’t mind volunteering. … We're just a little bit more experienced and mature and confident. … I think something that plays a role is the fact that we know that in a few months’ time we will be doctors, and we need to do it, so you start actually actively biting the bullet and trying to do it*

*[E]*

Students did not always recognise the opportunities for learning inherent in everyday clinical tasks.

*Some of the doctors on the ward rounds like literally just want us to tag along behind them to do the bloods and take it to the lab, or fill in the x-ray form, whenever they still had x-ray forms, take it to x-rays or whatever, and you don’t learn anything.*

[H]

*… at the end of the ward round you come to your patients and you are so bored out of your mind because this is the fourth hour of your ward round. So, that I find works better, where you are told listen, everybody needs to present one patient and make sure it’s at the beginning of the round*.

[FS]However, some students came to the realisation that they could be more active in their learning

*So basically bottom line there is still a great deal of self-responsibility in the clinical setting on the student yourself.*

[A]

*It’s like you are not just given information. You actually have to think for yourself as well. You have to find it, you have to create a response to this*

[H]

*I feel individually you need to learn to do this because one day you are going to be on your own, and there is not going to be anybody out there to help you, so you need to learn. So you need to take the opportunity yourself to do the procedures. But I don’t think all students feel that way, and I don’t think all students do that.*

[FS2]

Of interest to us was that despite well-articulated expectations that were not always met, students did not express a sense of agency to be able to shift their clinical experiences to meet these. The descriptions indicated a degree of passivity, a dependence on doctors to teach them and a low sense of agency. While they appreciated the value of responsibility for patient care, they were waiting for it to be given, rather than setting out to take this on. They referred to calibrating their learning by wanting the teachers to probe, question and check on them, rather than being able to ask for feedback, to self-assess or ask peers to assess. The “difficulty” of the clinical environment was aggravated by uncertainty, challenges in developing relationships with clinicians and a lack of clarity in their minds as to what clinicians’ responsibilities were with regards to teaching. Their approach to learning, seemed to be waiting to be taught, waiting to be given opportunities, wanting to be given the “right” answers, and wanting their days to be given structure.

As graduation looms, some students realise the need to be more assertive about ensuring they acquire the knowledge and skills necessary to be able to function independently in their internship.

## Discussion

The aim of this study was to understand students’ expectations and experiences of clinical teaching and what this means for strengthening faculty development initiatives for clinical teachers. Our initial analysis discovered a tension between students’ prior expectations and their experience of clinical teaching. With a view to exploring what this might mean for faculty development, we returned to the data to find how students resolved this tension. This led us to finding that a few of the students exercised agency in deciding to brave the discomfort of participating in clinical activities to become more active in their clinical learning. In this discussion, we explore the notion of personal agency, in particular the role that faculty development for clinical teachers could play in encouraging students to engage with clinical learning opportunities.

In the final phase of training, medical students encounter a liminal space between the structured, systematic, less ambiguous space of classroom learning (student) and the uncertain, chaotic and opportunistic space of workplace learning (intern). Successful transition across this space is an important part of the journey to becoming a clinician. In this study, students were interviewed just prior to graduation, thus having had exposure to a variety of clinical teachers in a number of different disciplines and a number of different clinical settings. Their descriptions of how clinicians approach teaching in the clinical environment differ little from experiences described in the literature on perceptions of clinical teaching [26, 27]. Students expected that clinical rotations would have the potential for them to apply their knowledge and test their procedural abilities in the environment where they would soon be exercising their professional practice and identity. However, they experienced the clinical environment as not supportive of clinical teaching, feeling that teaching is secondary to clinicians’ focus on patient care and that teaching was opportunistic, insufficiently structured and not skilfully delivered. Students appreciated that learning requires them to move out of their “comfort zone”, but seemed to persist in the idea of being recipients of teaching rather than becoming directors of their own learning.

We suggest that if students are to experience the learning space of the clinical environment, as one in which their expectations of participation can be met and their learning optimised, they may need assistance in developing their ability to engage. To facilitate this engagement they need to both understand their personal way of constructing knowledge, their beliefs about knowledge and knowing, and to develop their agentic capability. In understanding our findings in relation to the design of faculty development to strengthen clinical teaching, we draw on the work of Bandura, Billett and Dweck.

It seems that the students in this research largely utilised what Bandura [28] refers to as proxy agency where they depended on the clinicians to act in their best interest by directing their learning in ways that would enable the student to graduate. Social cognitive theory suggests that the intentional action which students take to make learning happen depends on their beliefs about what capacity they have to exercise control over the nature and quality of their life. Bandura [28] also refers to two other modes of exercising agency, namely direct personal agency and collective agency exercised through socially coordinative and interdependent effort. In terms of faculty development initiatives, it seems as though it may be worthwhile to consider ways in which the students’ direct personal agency could be encouraged by clinicians. In addition, the prospect of socially coordinative and interdependent agency speaks to students being enabled to join teams and the purposeful creation of environments conducive to learning where the interdependence of clinicians and students is recognised and utilised.

In this research students’ expressed expectations of participation in the clinical environment, but experienced that the affordances to do so were not always offered. This challenges faculty developers who are assisting clinicians to enable students’ learning for professional preparation, to include both the offering of affordances for student engagement, but also the promotion of students’ exercise of agency for participation. Billett’s socio-cultural lens [14] has taken Bandura’s work further by understanding that workplace learning occurs through participatory practices. Personal factors, that he refers to as engagement (how individuals elect to make use of the opportunities afforded them in the workplace), modify this participation and therefore the student’s construction of their learning.

Exercise of personal agency mitigates against the student merely being subjected to what is experienced in the workplace, enabling them to participate and construct their knowledge. Students supported to approach learning in this way are motivated because they see that engaged learning results in mastery of the knowledge and skills required of them when they graduate and join the workplace. Utilising achievement motivation and encouraging the student to develop a growth mindset [29], where intellect is viewed as not inherently fixed but able to be developed through practice, can be valuable skills for clinical teachers. It is more likely that personal agency will be exercised, enabling a student to participate in affordances that are offered.

The understanding of how to optimise students’ learning provided through the interplay of social cognitive, socio-cultural and achievement motivation theories supports the importance of clinicians strengthening their teaching by developing not only students’ proxy agency, but also their direct personal and collective agency. The question then arises as to how this could be done.

In their 2013 paper, Richards et al. [30] outline the five factors that support medical student agency and conclude by suggesting that “medical schools need to consider equipping their students with the necessary skills to engage effectively in their clinical learning” and that “students would benefit from the inclusion of the interdependent agentic capacities to enhance their learning experiences”. However, they do not suggest who should do the equipping! In our research, despite a low sense of agency, students expected and anticipated engagement. It seems clear that the role of the clinical teacher to not only offer engagement experiences (affordances), but also to foster students’ sense of agency is pivotal in maximising the potential of clinical learning. Therefore, we suggest that faculty development initiatives need to more intentionally incorporate these aspects when considering how to strengthen clinicians in their teaching role.

A limitation of this study is that the interviews elicited a dominance of unmet needs. As the focus of the discussions was on how clinical teaching could be improved, it is likely that this evoked responses about what students thought was not happening; exploration of the students’ positive experiences may have elicited practices that had the potential to be reinforced through faculty development offerings. These student participants were in the last two months of their programme; we did not establish the level of agency that students starting their clinical placements may have had. While we chose to use focus groups, individual interviews may have been able to generate even richer insights. Further research could valuably explore the most effective ways to equip clinicians with an understanding of student agency and how to nurture it. It would be useful to establish the most effective point(s) at which the development of student agency should be attended to.

The findings of this research illustrate the extent to which students’ expectations at this stage of their studies are for involvement as integral members of a clinical team. However, their experience is a struggle to position themselves for this involvement. Sometimes they even express resentment about having to engage in clinical work which takes them away from studying. This is despite recognising that it is exactly this involvement in the everyday tasks of the clinical team that will result in being invited to become a member of the team and that it is this participation that will assist both their learning and their preparation for the world of work – crossing the liminal space from classroom to bedside.

## Conclusions

In their 2008 AMEE guide, McLean et al. [31] suggest that in order to fulfil the mandate of medical education, faculty development must develop teachers who are genuinely committed to the holistic development of health care practitioners and to improving student learning. In considering how faculty developers can improve student learning, the perspectives of students in this research suggest that an important challenge is the need to foster personal agency in students. For learning in the workplace, clinical teachers may be best placed to meet this need. We therefore suggest that understanding the concept of student agency and its relationship to both the offering of appropriate affordances by clinicians and the acceptance of engagement by students, along with techniques and skills for developing students’ personal agency, are important additions to faculty development initiatives offered to clinical teachers. Faculty developers should consider incorporating ways in which clinicians can afford all students opportunities to take ownership of their learning and demonstrate agency in enhancing their participation in a workplace learning environment.

## Additional file

Additional file 1: Interview prompt questions. (DOCX 12 kb)
