# Supplementary material for: Designing faculty development: lessons learnt from a qualitative interpretivist study exploring students’ expectations and experiences of clinical teaching
Source: BMC Med Educ. 2019 Feb 7;19:49. doi: 10.1186/s12909-019-1480-7 (PMC6367744; doi:10.1186/s12909-019-1480-7)
Supplement: Supplementary file 1 — Interview prompt questions. (DOCX 12 kb) [file 12909_2019_1480_MOESM1_ESM.docx]

Additional File 1

Prompt questions

How do you experience clinical rotations; in particular, what do you identify as clinical teaching? What is the object of the exercise of clinical teaching? Why do we have clinical teaching? What makes it different from any of the other teaching that you ever had? How often do you get a chance to actually try it out under supervision? How often has somebody actually observed you? How do you know that you are seeing the things you should be seeing? Do you ever have a chance to watch somebody else doing a history or an examination?

What clinical teaching practices do you consider to have been of the greatest benefit to your learning? Anything that you have been exposed to that has really worked well for you in terms of learning? How often were you given responsibility? How does the teaching on ward rounds happen?

What criteria do you use to evaluate good clinical teaching? How do you recognise good clinical teaching, and what is it that you judge it against? If you could create the world’s best clinical teacher, what attributes would those be?

Any suggestions that you have for the improvement of clinical teaching? How do we help more clinicians to teach better? What is the one thing that you think could be done to make clinical teaching better? What would make it better for you?
